# Hematuria following stereotactic body radiation therapy (SBRT) for clinically localized prostate cancer

**DOI:** 10.1186/s13014-015-0351-6

**Published:** 2015-02-19

**Authors:** Marie K Gurka, Leonard N Chen, Aditi Bhagat, Rudy Moures, Joy S Kim, Thomas Yung, Siyuan Lei, Brian T Collins, Pranay Krishnan, Simeng Suy, Anatoly Dritschilo, John H Lynch, Sean P Collins

**Affiliations:** Department of Radiation Oncology, University of Louisville, Louisville, USA; Department of Radiation Medicine, Georgetown University Hospital, 3800 Reservoir Road, N.W, Washington D.C, 20007 USA; Department of Urology, Georgetown University Hospital, Washington, USA; Department of Radiology, Georgetown University Hospital, Washington, USA

## Abstract

**Background:**

Hematuria following prostate radiotherapy is a known toxicity that may adversely affect a patient’s quality of life. Given the higher dose of radiation per fraction using stereotactic body radiation therapy (SBRT) there is concern that post-SBRT hematuria would be more common than with alternative radiation therapy approaches. Herein, we describe the incidence and severity of hematuria following stereotactic body radiation therapy (SBRT) for prostate cancer at our institution.

**Methods:**

Two hundred and eight consecutive patients with prostate cancer treated with SBRT monotherapy with at least three years of follow-up were included in this retrospective analysis. Treatment was delivered using the CyberKnife® (Accuray) to doses of 35–36.25 Gy in 5 fractions. Toxicities were scored using the CTCAE v.4. Hematuria was counted at the highest grade it occurred in the acute and late setting for each patient. Cystoscopy findings were retrospectively reviewed. Univariate and multivariate analyses were performed. Hematuria-associated bother was assessed via the Expanded Prostate Index Composite (EPIC)-26.

**Results:**

The median age was 69 years with a median prostate volume of 39 cc. With a median follow-up of 48 months, 38 patients (18.3%) experienced at least one episode of hematuria. Median time to hematuria was 13.5 months. In the late period, there were three grade 3 events and five grade 2 events. There were no grade 4 or 5 events. The 3-year actuarial incidence of late hematuria ≥ grade 2 was 2.4%. On univariate analysis, prostate volume (p = 0.022) and history of prior procedure(s) for benign prostatic hypertrophy (BPH) (p = 0.002) were significantly associated with hematuria. On multivariate analysis, history of prior procedure(s) for BPH (p < 0.0001) and α_1A_ antagonist use (p = 0.008) were significantly associated with the development of hematuria.

**Conclusions:**

SBRT for prostate cancer was well tolerated with hematuria rates comparable to other radiation modalities. Patients factors associated with BPH, such as larger prostate volume, alpha antagonist usage, and prior history of procedures for BPH are at increased risk for the development of hematuria.

## Background

Radiation-induced hematuria is common after prostate cancer treatment due to the close proximity of the bladder and urethra to the prostate [1]. However, the incidence of clinically significant (≥ grade 2) late gross hematuria after conventionally fractionated external beam radiation therapy is generally < 5%. Hematuria commonly occurs within the first three years following treatment but may occur many years later [2-4]. In addition, hematuria is a common clinical problem even in those without a history of radiation treatment [5,6] and can be caused by urinary tract infections, urolithiasis, benign prostatic enlargement (BPH) and urologic malignancy. As hematuria can be a sign of serious genitourinary disease, the evaluation includes cystoscopy and imaging of the upper urinary tract. Approximately 20% of patients with gross hematuria are found to have a tumor of the urinary tract [6].

The risk of radiation-induced hematuria is dependent upon both the total radiation dose and the volume of the urethra/bladder neck in the high dose area [7]. Treatment related factors such as utilization of brachytherapy [8,9] or concurrent androgen deprivation therapy [10] may impact the risk of hematuria. Patient characteristics, such as a history of prior urethral procedures [10-12] and/or chronic anticoagulation therapy may increase an individual patient’s risk of clinically significant hematuria [13]. Whether hypofrationated radiation therapy is safe in these high risk populations is yet to be explored.

Clinical data suggest that hypofractionated radiation therapy may be radiobiologically favorable to smaller fraction sizes in prostate cancer radiotherapy [14]. The α/β for prostate cancer may be as low as 1.5 Gy [14]. If the α/β for prostate cancer is less than 3 Gy, which is generally the value accepted for late urinary complications, the linear-quadratic model predicts that delivering large radiation fraction sizes will result in improved local control with a similar rate of urinary complications. Early data for high dose rate brachytherapy monotherapy reveals that such regimens are effective without undue urinary toxicity [15].

Stereotactic body radiation therapy (SBRT) is a relatively new approach that is emerging as a standard treatment option for prostate cancer [16,17]. SBRT uses even larger daily fractions of radiation (7–9 Gy) to take advantage of this postulated radiobiological advantage. Emerging data suggest that this approach may provide similar clinical outcomes as other radiation modalities with high rates of biochemical control and low rates of grade 3 and higher toxicities [16,17]. Based on patient preference for a shorter treatment course, SBRT utilization is likely to increase as long as toxicity is acceptable. Here, we present our institutional hematuria rates following SBRT for clinically localized prostate cancer.

## Methods

### Patient selection

Georgetown University Hospital established its Prostate SBRT Program in 2006. As of December 2013, 700 prostate cancer patients have been treated with SBRT alone (monotherapy) or as a boost after conventionally fractionated external beam radiation therapy. A small subset of these patients also received androgen deprivation therapy (ADT) at the discretion of the treating radiation oncologist or the patient’s urologist. At the inception of our program, a prospective database was established to record baseline patient characteristics. At each follow-up visit, toxicity and quality of life data have also been prospectively collected and recorded. Patients eligible for this study were those who had SBRT monotherapy with or without ADT for clinically localized prostate cancer. Internal Review Board (IRB) approval was obtained for retrospective review of our database.

### SBRT treatment planning and delivery

**Figure 1:**
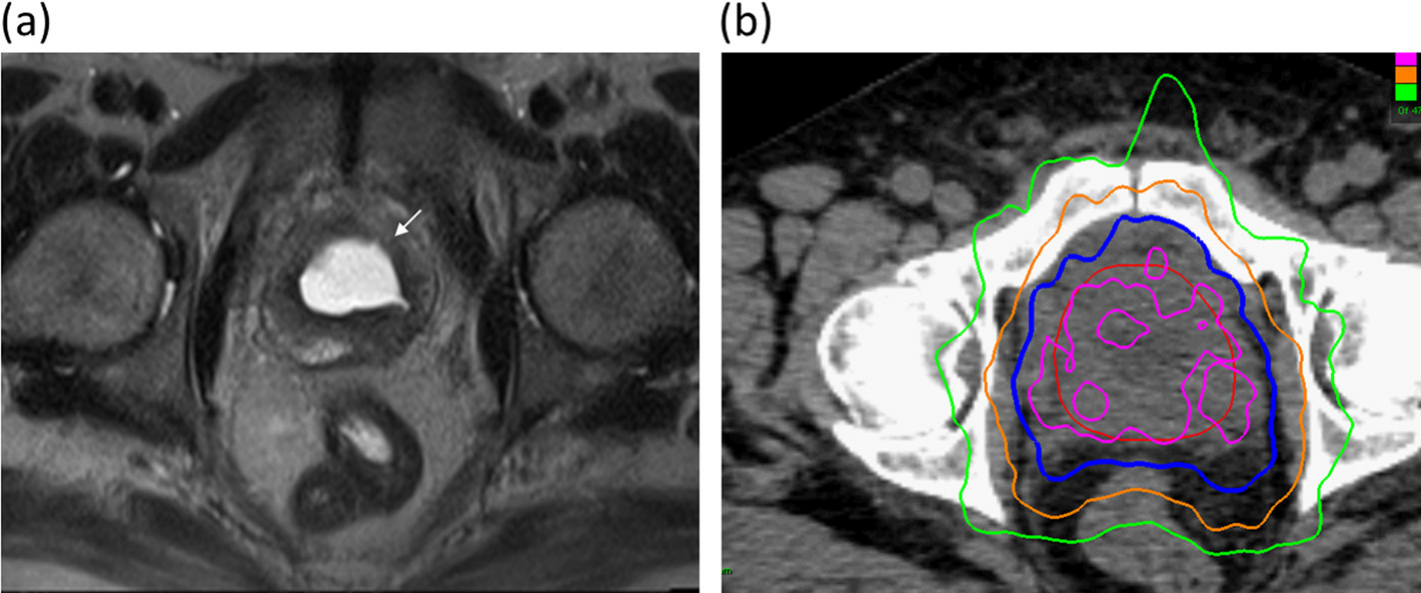
Treatment plan. (a) Axial T2-weighted MR image demonstrating central simple prostatectomy defect (arrow). (b) Treatment planning axial CT images demonstrating the prostate (red line). Isodose lines shown as follows: 115% of the prescription dose, pink line; 100% of the prescription dose, blue line: 75% of the prescription dose, orange line; and 50% of the prescription dose, green line.

SBRT treatment planning and delivery were conducted as previously described (Figure 1) [18,19]. Four gold markers were placed into the prostate 5–7 days prior to simulation. Patients were simulated with an empty bladder. Fused computed tomography (CT) and magnetic resonance (MR) images were used for treatment planning. The clinical target volume (CTV) included the prostate and the proximal seminal vesicles. Intrafraction image-guidance was employed to minimize the required PTV treatment margins [20]. The PTV equaled the CTV expanded 3 mm posteriorly and 5 mm in all other dimensions. The prescription dose was 35–36.25 Gy to the PTV delivered in five fractions of 7–7.25 Gy corresponding to a tumor EQD2 of approximately 85–90 Gy assuming an alpha/ beta ratio of 1.5. The prescription isodose line was limited to ≥ 75%, which limited the maximum prostatic urethra dose to 133% of the prescription dose. The bladder was contoured and evaluated with dose-volume histogram analysis during treatment planning using Multiplan (Accuray Inc., Sunnyvale, CA) inverse treatment planning. The empty bladder volume receiving 37 Gy was limited to < 10 cc in all patients. The bladder dose-volume histogram (DVH) goals were < 40% bladder volume receiving 50% of the prescribed dose and < 10% receiving 100% of the prescribed dose. To minimize the risk of local recurrence, the dose to the prostatic urethra was not constrained [21].

### Follow-up and statistical analysis

Hematuria was prospectively documented at follow-up visits using the National Cancer Institute (NCI) Common Toxicity Criteria (CTC) version 4.0. Acute hematuria was defined as experiencing toxicity within 3 months of radiation therapy. Late hematuria was defined as occurring at least 3 months after delivery of radiation therapy. If a hematuria episode occurred at a time point falling between follow-ups, it was counted at the later follow-up. Grade 1 represents minimal bleeding not requiring medications for symptom control. Grade 2 indicates hematuria requiring new medication (i.e. finasteride, aminocaproic acid). Grade 3 indicates hematuria requiring surgical intervention (i.e. coagulation, TURP). Hematuria evaluations were performed at the discretion of the patient’s urologist [5] and cystoscopy findings were retrospectively reviewed.

Patients completed the Expanded Prostate Cancer Index Composite (EPIC)-26 [22] before treatment and during routine follow-up visits one month after the completion of SBRT, every 3 months for the first year and then every 6 months for the second and third years. Bother due to hematuria was assessed via question 4c of the EPIC-26 (How big a problem, if any, has bleeding with urination been for you during the last four weeks?) [22]. The responses were grouped into three clinically relevant categories (no problem, very small-small problem and moderate to big problem).

Categorical demographic and disease related factors such as age, race, risk groups, procedures for benign prostate hypertrophy (BPH) (yes or no), baseline AUA scores, Charlson Cormobidity Index (CCI) at baseline, and anticoagulant usage (aspirin, clopidogrel, or warfarin) prior to treatment (yes or no) were compared using Chi-square analysis between patients reported hematuria during follow-up and those who did not. Multivariate analysis was performed using Cox’s proportional hazard regression model. All calculations were performed using SPSS v. 22 (IBM).

## Results

From February 2008 to August 2011, 208 prostate cancer patients were treated per our institutional SBRT monotherapy protocol (Table 1). The median follow-up was 48 months (range, 36–60). They were ethnically diverse with 45.2% being of non-Caucasian ancestry and a median age of 69 years (48–90). Comorbidities were common with 32.2% taking anticoagulants prior to treatment. The median prostate volume was 39 cc (11.6-138.7), 27.4% of patients utilized alpha-antagonists prior to SBRT and 11.5% had prior procedures for benign prostatic hyperplasia (BPH). By D’Amico classification, 82 patients were low-, 109 intermediate-, and 17 high-risk. Thirty patients (14.4%) also received androgen deprivation therapy. The majority of the patients (87.5%) were treated with 36.25 Gy in five 7.25 Gy fractions.

**Table 1:** **Baseline patient characteristics and treatment**

		**Patients (N = 208)**	**n**
**Age (y/o)**	Median 69 (48–90)		
	Age ≤ 60	12.5%	26
	60 < Age ≤ 70	46.2%	96
	Age > 70	41.3%	86
**Race**	White	54.8%	114
	Black	38.0%	79
	Other	7.2%	15
**Charlson Comorbidity Index**	CCI = 0	68.3%	142
	CCI = 1	22.6%	47
	CCI ≥ 2	9.1%	19
**Body Mass Index (BMI)**	Median 27.34 (15.02-44.96)		
	BMI ≥ 30	29.3%	61
**Partner Status**	Married or Partnered	76.4%	159
	Not Partnered	23.6%	49
**Employment Status**	Working	47.6%	99
	Not Working	52.4%	109
**Median Prostate Volume (cc)**	Median 38.6 (11.6-138.7) cc		208
**Baseline AUA Score**	Median 7 (0 – 34)		141
**Procedure for BPH**		11.5%	24
**α**_**1A**_ **inhibitor usage**		27.4%	57
**Anticoagulant usage**		32.2%	67
**Risk Groups (D’Amico’s)**	Low	39.4%	82
	Intermediate	52.4%	109
	High	8.2%	17
**ADT**		14.4%	30
**SBRT Dose**	36.25 Gy	87.5%	182
	35 Gy	12.5%	26

Baseline hematuria was uncommon. A total of 38 (18.3%) patients reported at least one episode of hematuria following SBRT. The median time to hematuria was 13.5 months (range, 5–22). Twenty nine patients reported a single episode (five of these were in the acute setting) and 9 patients reported multiple episodes. Eleven (5.4% of all patients) patients reported acute bleeding and 24 (11.8% of all patients) patients reported late bleeding. In the acute setting, there were two grade 2 toxicities. In the late period, there were five grade 2 events and three grade 3 events. There were no grade 4 or 5 events anytime during follow-up. No patient required a blood transfusion or hyperbaric oxygen. The 3-year actuarial incidence of late hematuria ≥ grade 2 was 2.4%. Hematuria had resolved in 95% of patients by the subsequent follow-up. Interestingly, of the grade 2–3 events, five patients had a history of transurethral resection of the prostate (TURP) or similar procedure prior to SBRT.

On univariate analysis, prostate volume (p = 0.022) and history of prior procedure(s) for BPH (p = 0.002) were significantly associated with hematuria. On multivariate analysis, history of prior procedure(s) for BPH (p ≤ 0.0001) and α_1A_ antagonist use (p = 0.008) were significantly associated with the development of hematuria (Table 2). No other baseline patient characteristics were significantly associated with hematuria following SBRT.

**Table 2:** **Impact of patient characteristics and treatment on hematuria incidence within three years post-SBRT**

**Factors**	***Univariate analysis***	***Multivariate analysis***
**Age**	**0.651**	**0.419**
**Race**	**0.523**	**0.917**
**D’Amico’s Risk Groups**	**0.805**	**0.122**
**Prostate Volume**	**0.022†**	**0.081**
**Initial PSA**	**0.546**	**0.804**
**Androgen Deprivation Therapy**	**0.507**	**0.507**
**Procedure for BPH prior to RT**	**0.002†**	<0.0001*
**Initial AUA**	**0.508**	**0.897**
α_**1A**_ **Antagonist usage**	**0.082**	0.008*
**Charlson Comorbidity Index**	**0.787**	**0.101**
**Anticoagulant usage**	**0.321**	**0.435**

†*significant in univariate analysis,* *significant in multivarariate analysis.

Nineteen patients had one or more cystoscopies during the follow-up period. The median interval from completion of SBRT to cystoscopy was 24 months (range, 5–42 months). Radiation was the causative factor in 11 (60%) of these patients. The most common radiation associated findings on cystoscopy were hyperemia of the bladder neck/prostatic urethra (Table 3). Minor coagulation was performed in 2 patients. Three patients were found to have a bladder cancer at 18, 30, and 36 months after SBRT.

**Table 3:** **Patients with hematuria who underwent cystoscopy**

**Patient #**	**Pre-RT TURP**	**Anti-coagulant usage**	**Hematuria (months post-RT)**	**Cystoscopy finding**	**Etiology of hematuria**
1			42	Hyperemic prostatic urethra; bleeding from prostatic urethra	Radiation
2			18, 30, 36	Bladder cancer	Bladder cancer
3			24	Hyperemic bladder neck	Radiation
4	Yes		18	Necrotic tissue in TURP defect, hyperemic bladder neck	Radiation
5			18	Normal	
6	Yes	Yes	12	Varices of the prostatic urethra	Radiation
7	Yes		32	Hyperemic bladder neck	Radiation
8	Yes	Yes	5	Hyperemic prostatic urethra and bladder neck	Radiation
9		Yes	11	Bleeding from prostatic urethra	Radiation
10	Yes	Yes	24	Hyperemic prostatic urethra and bladder neck	Radiation
11			9, 24	Normal	Kidney stone
12			30	Bladder cancer	Bladder cancer
13			6, 18	Hyperemic bladder neck and bladder stone	Bladder stone
14			15	Normal	Kidney stone
15			36	Bladder cancer	Bladder cancer
16		Yes	27	Normal	
17	Yes	Yes	3, 6, 9, 12	Necrotic tissue in TURP defect; hyperemic bladder neck and prostatic urethra	Radiation
18	Yes		1, 3, 6, 9, 12, 18	hyperemic bladder neck; bleeding from prostatic urethra	Radiation
19	Yes	Yes	24	Normal	Kidney stone

At baseline, 3% of our cohort reported some level of annoyance due to hematuria; however, only one patient felt it was a moderate to big problem (Table 4). Hematuria bother increased following treatment and peaked at 9 months post treatment with 2.7% of patients reporting that it was a moderate to big problem (Table 4). Hematuria bother declined quickly and returned to baseline by three years after SBRT with 1.3% of men feeling that their hematuria was a moderate to big problem three years post-SBRT (Table 4).

**Table 4:** **Hematuria bother following SBRT for prostate cancer (patient-reported responses to question 4c of the EPIC-26)**

	**Start**	**1**	**3**	**6**	**9**	**12**	**18**	**24**	**30**	**36**
No Problem	96.6%	96.5%	97.0%	97.3%	93.1%	96.1%	96.4%	97.2%	96.5%	97.5%
Very Small-Small Problem	2.9%	2.5%	1.5%	2.1%	4.8%	2.8%	3.0%	2.8%	2.9%	1.3%
Moderate-Big Problem	0.5%	1.0%	2.0%	1.1%	2.7%	1.7%	1.2%	0.6%	1.2%	1.3%
Total N=	207	202	200	188	188	181	167	176	172	157

## Discussion

To our knowledge, this is the first study to report cystoscopy findings and the incidence of hematuria following SBRT monotherapy for prostate cancer. Hematuria was uncommon at any time point and transient in most instances. The prevalence of patient reported hematuria peaked at 9 months and resolved spontaneously without recurrence. Despite the high biochemical equivalent dose delivered by SBRT in this series, these results are similar to conventionally fractionated EBRT and brachytherapy with published studies reporting rates of hematuria that range from 2.6% - 14% [23-26,9].

The etiology of late hematuria following prostate cancer radiation therapy is multi-factorial [27]. Urolithiasis, benign prostatic enlargement (BPH) and urologic malignancy were the most common non-radiation associated causes in our patients. Previous studies have also reported that hematuria due to radiation therapy is associated with pretreatment factors related to BPH such as a high AUA score, large prostate volume, and pre-radiation TURP [28]. Our data also supports these conclusions since history of a TURP or similar procedure, α_1A_ antagonist use, and increasing prostate volume were significantly associated with the development of post-radiation hematuria. One patient who experienced a grade 3 hematuria had a history of benign prostatic hypertrophy with a large prostate and two prior TURP procedures prior to receiving SBRT [18].

It has been well documented that pre-treatment TURP, especially if done very close to radiation, increases the risk of other late urinary toxicities such as stricture and incontinence [11,29,30]. This is thought to be caused by devascularization of the urethra after TURP and the lessened ability of the mucosa to repair sublethal radiation damage [30]. In addition, the anatomy of the prostate base is altered after a TURP resulting in broadening of the base; therefore, the treatment volume at the bladder interface may include a larger part of the bladder neck in the PTV [29].

Patients in our study with hematuria secondary to radiation, who underwent cystoscopy, often had some degree of bladder neck and/or prostatic urethra edema and hypervascularity supporting the above hypotheses. These cystoscopy findings have also been observed in patients with hematuria after prostate brachytherapy [25]. In an effort to decrease the incidence of bladder neck and prostatic urethra pathology, we have reduced the central dose in our relatively inhomogenous plans [18]. It is expected, that with these modifications, the risk of hematuria will decline.

Interestingly, in our study, we did not find an association between anticoagulation usage and hematuria as previously reported by others [13]. One possible explanation is that we also included aspirin (low or high dose), along with clopidogrel and warfarin in the analysis, whereas most only include the latter two. Aspirin, especially low dose, may not predispose patients to bleeding as strongly as warfarin or clopidogrel and may explain the discrepancy.

In this study, the PTV included the prostate and proximal seminal vesicles with margin regardless of risk group, as do many [31-34]. This is in contrast to the Radiation Therapy Oncology Group (RTOG), which recommends inclusion of the prostate only with margin in the PTV for low risk prostate cancer. How the inclusion of the proximal seminal vesicles affects the risk of hematuria is unknown. A prior publication has demonstrated that increasing the volume of the seminal vesicles included in the PTV would only have a moderate increase in normal tissue complication probability (NTCP) [35].

Hematuria following prostate cancer treatment is also a quality of life issue [36]. Bother is defined as the degree of interference or annoyance caused by a symptom [37,38]. In this series, moderate to severe bother with hematuria was uncommon. During the first two years following SBRT, 1-2% of men reported hematuria bother as a moderate to big problem at any given follow-up. This change is comparable to with conventionally fractionated radiation therapy and brachytherapy [36,39].

Three patients were diagnosed with bladder tumors (1.4% of patients) at a median time of 30 months. These results are consistent with those reported following conventionally fractionated external radiation therapy and brachytherapy [40,41]. Likely, these early bladder tumors were discovered due to increased surveillance and were unlikely caused by SBRT due to the short interval to development from treatment. The cystoscopy reports were available for two of the three patients. One patient had a non-muscle invasive tumor of the right lateral wall. The second patient developed recurrent superficial papillary tumors throughout the bladder. Nonetheless, our results emphasize the importance of cancer screening in patients who report gross hematuria following SBRT. Longer follow-up will be required to identify the true incidence of SBRT-associated bladder tumors.

Our study has several limitations. We present the self-reported hematuria incidence. As most men did not undergo regular urinalysis during follow-up, the true incidence of post-SBRT hematuria may be underestimated in this study. Likewise, most hematuria events were transient and associated bother may have been missed due to the timing of questionnaire administration [42]. Furthermore, only a subset of patients underwent cystoscopy. Therefore, the cause of hematuria is not known for all patients who reported it. In the future we plan to present a DVH analysis examining the association between urethral and bladder doses with hematuria in patients where radiation was the causative factor on cystoscopy. It is also possible that the cause of hematuria could differ between the acute and late setting; however, due to the low number of patients with isolated acute hematuria (n = 5) a meaningful statistical analysis could not be performed. And lastly, the median follow up in this group of patients is relatively short, and additional hematuria events could potentially be seen with longer follow up [3].

## Conclusions

SBRT for clinically localized prostate cancer was well tolerated with hematuria rates comparable to conventionally fractionated external radiotherapy and brachytherapy. Patients factors associated with BPH, such as larger prostate volume, α_1A_ antagonist use and prior history of procedures for BPH are at increased risk for the development of hematuria. Less than 5% of men felt post-treatment hematuria was a moderate to big problem at any time point during follow-up. These results need to be confirmed with longer follow-up.

## Abbreviations

ADT: Androgen deprivation therapy
CT: Computed tomography
CTC: Common toxicity criteria
CTV: Clinical target volume
DVH: Dose-volume histogram
EQD2: Equivalent dose in 2-Gy fractions
EPIC: Expanded prostate index composite
GTV: Gross target volume
Gy: Gray
IMRT: Intensity modulated radiation therapy
IRB: Internal review board
PTV: Planning target volume
QoL: Quality of life
MR: Magnetic resonance
NCI: National Cancer Institute
SD: Standard deviation
SBRT: Stereotactic body radiation therapy

## References

[1] Marks LB, Carroll PR, Dugan TC, Anscher MS (1995). The response of the urinary bladder, urethra, and ureter to radiation and chemotherapy. Int J Radiat Oncol Biol Phys.

[2] Mathieu R, Arango JD, Beckendorf V, Delobel JB, Messai T, Chira C (2014). Nomograms to predict late urinary toxicity after prostate cancer radiotherapy. World J Urol.

[3] Eifel PJ, Levenback C, Wharton JT, Oswald MJ (1995). Time course and incidence of late complications in patients treated with radiation therapy for FIGO stage IB carcinoma of the uterine cervix. Int J Radiat Oncol Biol Phys.

[4] Lawton CA, Won M, Pilepich MV, Asbell SO, Shipley WU, Hanks GE (1991). Long-term treatment sequelae following external beam irradiation for adenocarcinoma of the prostate: analysis of RTOG studies 7506 and 7706. Int J Radiat Oncol Biol Phys.

[5] Margulis V, Sagalowsky AI (2011). Assessment of hematuria. Med Clin North Am.

[6] van der Molen AJ, Hovius MC (2012). Hematuria: a problem-based imaging algorithm illustrating the recent Dutch guidelines on hematuria. AJR Am J Roentgenol.

[7] Viswanathan AN, Yorke ED, Marks LB, Eifel PJ, Shipley WU (2010). Radiation dose-volume effects of the urinary bladder. Int J Radiat Oncol Biol Phys.

[8] Leapman MS, Hall SJ, Stone NN, Stock RG (2013). Haematuria after prostate brachytherapy. BJU Int.

[9] Buckstein M, Carpenter TJ, Stone NN, Stock RG (2013). Long-term outcomes and toxicity in patients treated with brachytherapy for prostate adenocarcinoma younger than 60 years of age at treatment with minimum 10 years of follow-up. Urology.

[10] Zapatero A, Garcia-Vicente F, Sevillano D, Martin de Vidales C, Ferrer C, Torres JJ (2008). Is hormone therapy a protective factor for late hematuria after high-dose radiotherapy in prostate cancer?. Urology.

[11] Devisetty K, Zorn KC, Katz MH, Jani AB, Liauw SL (2010). External beam radiation therapy after transurethral resection of the prostate: a report on acute and late genitourinary toxicity. Int J Radiat Oncol Biol Phys.

[12] Sandhu AS, Zelefsky MJ, Lee HJ, Lombardi D, Fuks Z, Leibel SA (2000). Long-term urinary toxicity after 3-dimensional conformal radiotherapy for prostate cancer in patients with prior history of transurethral resection. Int J Radiat Oncol Biol Phys.

[13] Choe KS, Jani AB, Liauw SL (2010). External beam radiotherapy for prostate cancer patients on anticoagulation therapy: how significant is the bleeding toxicity?. Int J Radiat Oncol Biol Phys.

[14] Fowler JF (2005). The radiobiology of prostate cancer including new aspects of fractionated radiotherapy. Acta Oncol.

[15] Demanes DJ, Martinez AA, Ghilezan M, Hill DR, Schour L, Brandt D (2011). High-dose-rate monotherapy: safe and effective brachytherapy for patients with localized prostate cancer. Int J Radiat Oncol Biol Phys.

[16] King CR, Collins S, Fuller D, Wang PC, Kupelian P, Steinberg M (2013). Health-related quality of life after stereotactic body radiation therapy for localized prostate cancer: results from a multi-institutional consortium of prospective trials. Int J Radiat Oncol Biol Phys.

[17] King CR, Freeman D, Kaplan I, Fuller D, Bolzicco G, Collins S (2013). Stereotactic body radiotherapy for localized prostate cancer: pooled analysis from a multi-institutional consortium of prospective phase II trials. Radiother Oncol.

[18] Chen LN, Suy S, Uhm S, Oermann EK, Ju AW, Chen V (2013). Stereotactic body radiation therapy (SBRT) for clinically localized prostate cancer: the Georgetown University experience. Radiat Oncol.

[19] Lei S, Piel N, Oermann EK, Chen V, Ju AW, Dahal KN (2011). Six-dimensional correction of intra-fractional prostate motion with CyberKnife stereotactic body radiation therapy. Front Oncol.

[20] Xie Y, Djajaputra D, King CR, Hossain S, Ma L, Xing L (2008). Intrafractional motion of the prostate during hypofractionated radiotherapy. Int J Radiat Oncol Biol Phys.

[21] Vainshtein J, Abu-Isa E, Olson KB, Ray ME, Sandler HM, Normolle D (2012). Randomized phase II trial of urethral sparing intensity modulated radiation therapy in low-risk prostate cancer: implications for focal therapy. Radiat Oncol.

[22] Wei JT, Dunn RL, Litwin MS, Sandler HM, Sanda MG (2000). Development and validation of the expanded prostate cancer index composite (EPIC) for comprehensive assessment of health-related quality of life in men with prostate cancer. Urology.

[23] Barnett GC, De Meerleer G, Gulliford SL, Sydes MR, Elliott RM, Dearnaley DP (2011). The impact of clinical factors on the development of late radiation toxicity: results from the Medical Research Council RT01 trial (ISRCTN47772397). Clin Oncol.

[24] De Langhe S, De Meerleer G, De Ruyck K, Ost P, Fonteyne V, De Neve W, et al. Integrated models for the prediction of late genitourinary complaints after high-dose intensity modulated radiotherapy for prostate cancer: Making informed decisions. Radiotherapy and oncology. 2014 [Epub ahead of print] 10.1016/j.radonc.2014.04.005 24951017

[25] Barker J, Wallner K, Merrick G (2003). Gross hematuria after prostate brachytherapy. Urology.

[26] Mohammed N, Kestin L, Ghilezan M, Krauss D, Vicini F, Brabbins D (2012). Comparison of acute and late toxicities for three modern high-dose radiation treatment techniques for localized prostate cancer. Int J Radiat Oncol Biol Phys.

[27] Grise P, Thurman S (2001). Urinary incontinence following treatment of localized prostate cancer. Cancer Control.

[28] Ishiyama H, Hirayama T, Jhaveri P, Satoh T, Paulino AC, Xu B (2014). Is there an increase in genitourinary toxicity in patients treated with transurethral resection of the prostate and radiotherapy?: a systematic review. Am J Clin Oncol.

[29] Heemsbergen WD, Al-Mamgani A, Witte MG, van Herk M, Pos FJ, Lebesque JV (2010). Urinary obstruction in prostate cancer patients from the Dutch trial (68 Gy vs. 78 Gy): relationships with local dose, acute effects, and baseline characteristics. Int J Radiat Oncol Biol Phys.

[30] Seymore CH, el-Mahdi AM, Schellhammer PF (1986). The effect of prior transurethral resection of the prostate on post radiation urethral strictures and bladder neck contractures. Int J Radiat Oncol Biol Phys.

[31] Freeman DE, King CR (2011). Stereotactic body radiotherapy for low-risk prostate cancer: five-year outcomes. Radiat Oncol.

[32] Katz A, Kang J (2014). Stereotactic body radiotherapy with or without external beam radiation as treatment for organ confined high-risk prostate carcinoma: a six year study. Radiat Oncol.

[33] Spratt DE, Pei X, Yamada J, Kollmeier MA, Cox B, Zelefsky MJ (2013). Long-term survival and toxicity in patients treated with high-dose intensity modulated radiation therapy for localized prostate cancer. Int J Radiat Oncol Biol Phys.

[34] ACR Appropriateness Criteria® External Beam Radiation Therapy Treatment Planning for Clinically Localized Prostate Cancer. [https://acsearch.acr.org/docs/69396/Narrative/].

[35] Gluck I, Vineberg KA, Ten Haken RK, Sandler HM (2009). Evaluating the relationships between rectal normal tissue complication probability and the portion of seminal vesicles included in the clinical target volume in intensity-modulated radiotherapy for prostate cancer. Int J Radiat Oncol Biol Phys.

[36] Sanda MG, Dunn RL, Michalski J, Sandler HM, Northouse L, Hembroff L (2008). Quality of life and satisfaction with outcome among prostate-cancer survivors. N Engl J Med.

[37] Litwin MS, Gore JL, Kwan L, Brandeis JM, Lee SP, Withers HR (2007). Quality of life after surgery, external beam irradiation, or brachytherapy for early-stage prostate cancer. Cancer.

[38] Gore JL, Gollapudi K, Bergman J, Kwan L, Krupski TL, Litwin MS (2010). Correlates of bother following treatment for clinically localized prostate cancer. J Urol.

[39] Hoppe BS, Michalski JM, Mendenhall NP, Morris CG, Henderson RH, Nichols RC (2014). Comparative effectiveness study of patient-reported outcomes after proton therapy or intensity-modulated radiotherapy for prostate cancer. Cancer.

[40] Zelefsky MJ, Housman DM, Pei X, Alicikus Z, Magsanoc JM, Dauer LT (2012). Incidence of secondary cancer development after high-dose intensity-modulated radiotherapy and image-guided brachytherapy for the treatment of localized prostate cancer. Int J Radiat Oncol Biol Phys.

[41] Leapman MS, Stock RG, Stone NN, Hall SJ (2014). Findings at cystoscopy performed for cause after prostate brachytherapy. Urology.

[42] Litwin MS, McGuigan KA (1999). Accuracy of recall in health-related quality-of-life assessment among men treated for prostate cancer. J Clin Oncol.

